# Trends in methylphenidate dispensing and regional inequalities among children and adolescents in France around the 2021 prescription reform: a repeated cross-sectional ecological analysis of national data (2014–2024)

**DOI:** 10.1186/s12889-026-28213-3

**Published:** 2026-06-26

**Authors:** Edouard Guez, Judith Van der Waerden, David Cohen, Xavier Benarous

**Affiliations:** 1 Department of Child and Adolescent Psychiatry, Pitié-Salpêtrière Hospital, AP-HP.Sorbonne University, 83 boulevard de l’Hôpital, Paris, 75013 France; 2 Pierre Louis Institute of Epidemiology and Public Health (iPLesp), team Social Epidemiology, Sorbonne University, INSERM UMR-S1136, Mental Health and Addictions (ESSMA), Paris, 75012 France; 3 Institute for Intelligent Systems and Robotics, Sorbonne University CNRS UMR 7222, Paris, France

**Keywords:** Methylphenidate, Attention-deficit/hyperactivity disorder, Child and adolescent psychiatry, Prescription reform, Regional disparities, France

## Abstract

**Background:**

In 2021, France broadened methylphenidate prescribing conditions by allowing treatment initiation outside hospital-based specialist settings in children and adolescents. This regulatory change occurred during the COVID-19 period and alongside broader changes in attention-deficit/hyperactivity disorder (ADHD) recognition and care organization, making its specific effect difficult to disentangle from concurrent contextual factors. We therefore examined national trends in methylphenidate dispensing and regional inequalities in France over 2014–2024, interpreting the reform and pandemic period as contextual factors rather than isolated causal exposures.

**Methods:**

We conducted a repeated cross-sectional ecological study using annual region-level aggregated data from OPENMEDIC and INSEE for individuals aged 0–19 years in France from 2014 to 2024. OPENMEDIC records dispensed boxes rather than treatment initiations or treated individuals. Pre- and post-periods were defined as 2014–2019 and 2022–2024; 2020–2021 were treated as transition years. The primary outcome was annual methylphenidate dispensations per 1,000 inhabitants. Secondary outcomes included methylphenidate as a proportion of all psychotropic dispensations and regional inequality indices. All analyses were interpreted descriptively.

**Results:**

Methylphenidate dispensations per 1,000 inhabitants aged 0–19 years increased across all 13 French regions in 2022–2024 compared with 2014–2019. Nationally, dispensations rose from 0.058 per 1,000 in 2014 to 0.189 per 1,000 in 2024, corresponding to a 3.3-fold increase. In descriptive national and panel analyses, post-2022 slopes were steeper than pre-period slopes, whereas immediate level-change estimates were imprecise. The national proportion of methylphenidate among all psychotropic dispensations increased from 6.30% in 2014–2019 to 9.09% in 2022–2024. Regional inequality metrics showed no clear convergence.

**Conclusions:**

In this nationwide ecological study based on annually aggregated data, methylphenidate dispensing more than tripled in France between 2014 and 2024, with steeper post-2022 trends, while regional disparities remained observable and showed no clear convergence. Because the 2021 prescription reform coincided with the COVID-19 period and other changes in ADHD care, these findings should be interpreted as descriptive population-level trends rather than evidence of a specific reform effect.

**Supplementary Information:**

The online version contains supplementary material available at https://doi.org/10.1186/s12889-026-28213-3.

## Introduction

International estimates reveal substantial cross-national variation in methylphenidate treatment rates among children and adolescents across high-income countries. These rates range from approximately 6% in the United States [1, 2], 3–4% in the Netherlands [3], 2–3% in the United Kingdom [4], with lower values around 2% in Germany [5] and 1.6% Denmark [6]. In contrast, France remains among the lowest prescribers worldwide, with rates ranging from 0.4 to 0.7% [7, 8], similar to those reported in China (< 0.1%) [9]. These cross-national comparisons refer specifically to methylphenidate and should not be interpreted as direct comparisons of overall pharmacotherapy, as the relative use of other stimulants and non-stimulants varies across countries and over time.

Given that attention-deficit/hyperactivity disorder (ADHD) symptoms frequently lead to substantial functional impairments for affected children and their families, methylphenidate is recommended as the first-line pharmacological treatment in all major guidelines [10, 11]. Availability of ADHD stimulants varies across countries, and methylphenidate remains the only available stimulant in some settings while coexisting with other licensed stimulants in others [12, 13].

From the early 2000s to the mid-2010s, methylphenidate dispensing increased sharply in several high-income countries, with the highest prevalence in the US (up to 8.8% among 10-14-year-olds by 2012) and the Netherlands (7.1% in the same age group) [3]. France exhibited much lower but continuous growth, reaching 0.61–0.75% in 2019 [14], while Asian countries, notably China, remained below 0.1% [9]. After 2015, several European countries - most notably the Netherlands - reported declines linked to policy reforms (e.g., the Dutch Youth Act), shifting diagnostic practices, and concerns about medicalization [15]. In contrast, the United States continued to show rising stimulant prescriptions through 2021, including a modest increase in methylphenidate dispensing among children and adolescents, followed by a pandemic-related dip and subsequent rebound [16–18]. Throughout this period, methylphenidate remained the most prescribed ADHD medication worldwide, though alternative stimulants and non-stimulants expanded, especially in North America [16, 19]. Boys consistently exhibited higher dispensing rates than girls, with proportionally faster recent increases observed among adolescent girls in some settings [15, 19]. These variations appear multifactorial and context-dependent, reflecting improved recognition of ADHD, changes in diagnostic practices, service organization, professional and public awareness, and broader sociocultural factors [20]. At the same time, concerns have been raised that rising prescribing may also reflect overdiagnosis, medicalization, or treatment of less severe presentations in some contexts [21].

Cross-national variation is only partly attributable to ADHD prevalence [22]. Differences in clinical practice patterns, diagnostic cultures, thresholds for impairment, and regulatory frameworks also contribute to considerable heterogeneity [10, 23–26]. Social inequalities further compound this variability: children from disadvantaged backgrounds are more likely to receive ADHD medication once diagnosed, suggesting social disparities in clinical management rather than purely epidemiological differences [27, 28]. Access to ADHD treatment, therefore, represents a major public health issue, as social adversity and inequities in health systems may shape attention, behavioral, and emotional development [29, 30]. Patterns of methylphenidate dispensations may provide an indirect and partial indicator of access to pharmacological ADHD care, although they do not capture diagnosis quality, treatment appropriateness, or access to non-pharmacological interventions [31].

In France, child and adolescent psychiatric care is usually free in public facilities and sometimes available in private practice, with consultation costs partly covered by national health insurance and partly by private insurance. Methylphenidate-based medications are therefore fully reimbursed. Despite this universal coverage, substantial regional inequalities persist in access to ADHD treatment [32, 33]. This variation may be partly related to medical demographic disparities, specialist accessibility, and differences in care trajectories. In a French national survey, diagnostic and treatment delays varied according to professional background and specialist accessibility, underscoring how healthcare resources and territorial organization shape ADHD care pathways [34]. These territorial inequalities in psychiatrist availability persist nationwide: the French Ministry of Health’s 2018–2022 National Health Strategy reported a nineteen-fold difference in access to psychiatrists between territories [35]. Such imbalances have tangible clinical consequences in child and adolescent psychiatry; access to specialized care and evidence-based therapeutic options is influenced by social adversity and shortages of healthcare professionals [36]. A recent analysis of the *Système National des Données de Santé* (SNDS) [37] also reported rising methylphenidate use in autistic youth, suggesting possible systemic drivers of prescribing beyond ADHD.

On September 13, 2021, the French National Agency for the Safety of Medicines and Health Products (ANSM) broadened methylphenidate prescribing conditions by allowing initial prescriptions by specialists in neurology, psychiatry, or pediatrics, whether in hospitals or private practice; renewals remained possible by any physician within one year [38]. Before this reform, initial methylphenidate prescriptions could only be provided by hospital-based specialists. Because OPENMEDIC does not distinguish treatment initiation from continuation or renewal, this study could not directly assess the specific target of the reform. This regulatory change was intended to facilitate access to pharmacological treatment, particularly in areas with limited access to hospital-based specialists. However, it occurred during the COVID-19 period and alongside broader changes in ADHD recognition, service organization, and public policies, making it difficult to isolate its specific contribution using annually aggregated dispensing data.

The objective of this study was therefore to characterize national trends in methylphenidate dispensing and regional inequalities in France from 2014 to 2024, in the context of the September 2021 national regulatory reform that broadened prescribing conditions and the COVID-19 period. Specifically, we assessed (a) whether methylphenidate dispensing intensity increased nationally over time, and (b) whether regional disparities in methylphenidate dispensing-summarized using methylphenidate dispensations relative to all psychotropic dispensations-changed over the same period. This assessment takes place within a broader context in which multiple, interrelated policy and organizational initiatives have sought to improve ADHD care in France, which limits the extent to which observed changes can be attributed to a single measure [39, 40]. The COVID-19 pandemic also coincided with the policy period and likely affected healthcare use and prescribing patterns [41–43]. Therefore, our analyses were not designed to estimate the causal effect of the reform itself or to evaluate changes in treatment initiation specifically, but rather to provide a transparent description of population-level dispensing trends and persistent spatial inequalities using nationwide open-access dispensing data from OPENMEDIC, an aggregated extract derived from the French National Health Data System [37, 44]. We focus on spatial inequalities as a public health concern in this population and we note that socioeconomic adversity is associated with ADHD and related comorbidities such as disruptive behavior disorders [45]. Based on international trends and the direction of the regulatory change, we expected methylphenidate dispensing to increase over time [4, 9, 46–52], while we did not specify an a priori direction for changes in regional inequalities.

## Methods

### Data sources

We conducted a repeated cross-sectional ecological study using publicly available, aggregated annual data from France’s National Health Insurance outpatient drug dispensation database (OPENMEDIC; https://assurance-maladie.ameli.fr/etudes-et-donnees/open-medic-base-complete-depenses-medicaments) and population estimates from the National Institute of Statistics and Economic Studies (INSEE; https://www.insee.fr/fr/statistiques/8331297). OPENMEDIC records all reimbursed outpatient dispensations in France, excluding in-hospital dispensing. For each calendar year, we extracted methylphenidate dispensations (ATC5: N06BA04) and all psychotropic dispensations (ATC3: N03A, N04A, N04B, N05A, N05B, N05C, N06A, N06B, N07A, N07B). Because of database limitations, classes N07C and N07X were excluded. OPENMEDIC counts dispensed boxes rather than treated individuals or new treatment initiations; therefore, all study indicators reflect dispensing intensity rather than treatment prevalence or incidence. As an annually aggregated database, it does not allow patient-level analyses, including the number of treated individuals, incident versus prevalent use, or monthly analyses accounting for seasonality in methylphenidate dispensing. Annual regional population counts aged 0–19 years were obtained from INSEE and used to compute per-capita dispensing rates and population-weighted estimates. OPENMEDIC is an open-access, annually aggregated extract derived from the French National Health Data System.

### Population and geographic unit

The study population comprised individuals aged 0–19 years residing in France (mainland and overseas) over the period 2014–2024. This age range was used because publicly available OPENMEDIC aggregated data and INSEE regional population denominators are available in this age grouping, and age-subgroup disaggregation was not possible. We acknowledge that methylphenidate is not licensed for children under 6 years in Europe [12], and that clinical practice is generally more conservative in this age group. Therefore, rates expressed per 1,000 inhabitants aged 0–19 years should be interpreted as population-level dispensing intensity using the available denominator, rather than as age-specific treatment rates among clinically eligible children. The geographic unit of analysis was France’s 13 administrative regions (12 on the mainland plus 1 overseas grouping in OPENMEDIC), which are routinely used for national health statistics and regional health planning. This level of aggregation captures broad territorial variation in healthcare workforce distribution and service organization, while remaining compatible with publicly available OPENMEDIC data. All analyses were performed at the region-year level and at the national level by summing across regions.

### Study periods

To reduce potential contamination by pandemic-related disruptions and the implementation year of the 2021 methylphenidate prescription reform, we defined a pre-period (2014–2019) and a post-period (2022–2024). The years 2020 and 2021 were treated as transition years and were excluded from primary pre/post comparisons and from the main interrupted time-series models.

### Outcomes

The primary outcome was annual methylphenidate dispensations per 1,000 inhabitants aged 0–19 years, computed as 1000 × (regional MPH dispensations / regional population aged 0–19). Secondary outcomes were the proportion of methylphenidate (MPH) among all psychotropic (PT) dispensations (MPH/PT) and the absolute number of methylphenidate dispensations, the latter used for exploratory correlation analyses with structural regional characteristics.

### Statistical analysis

**Fig. 1:**
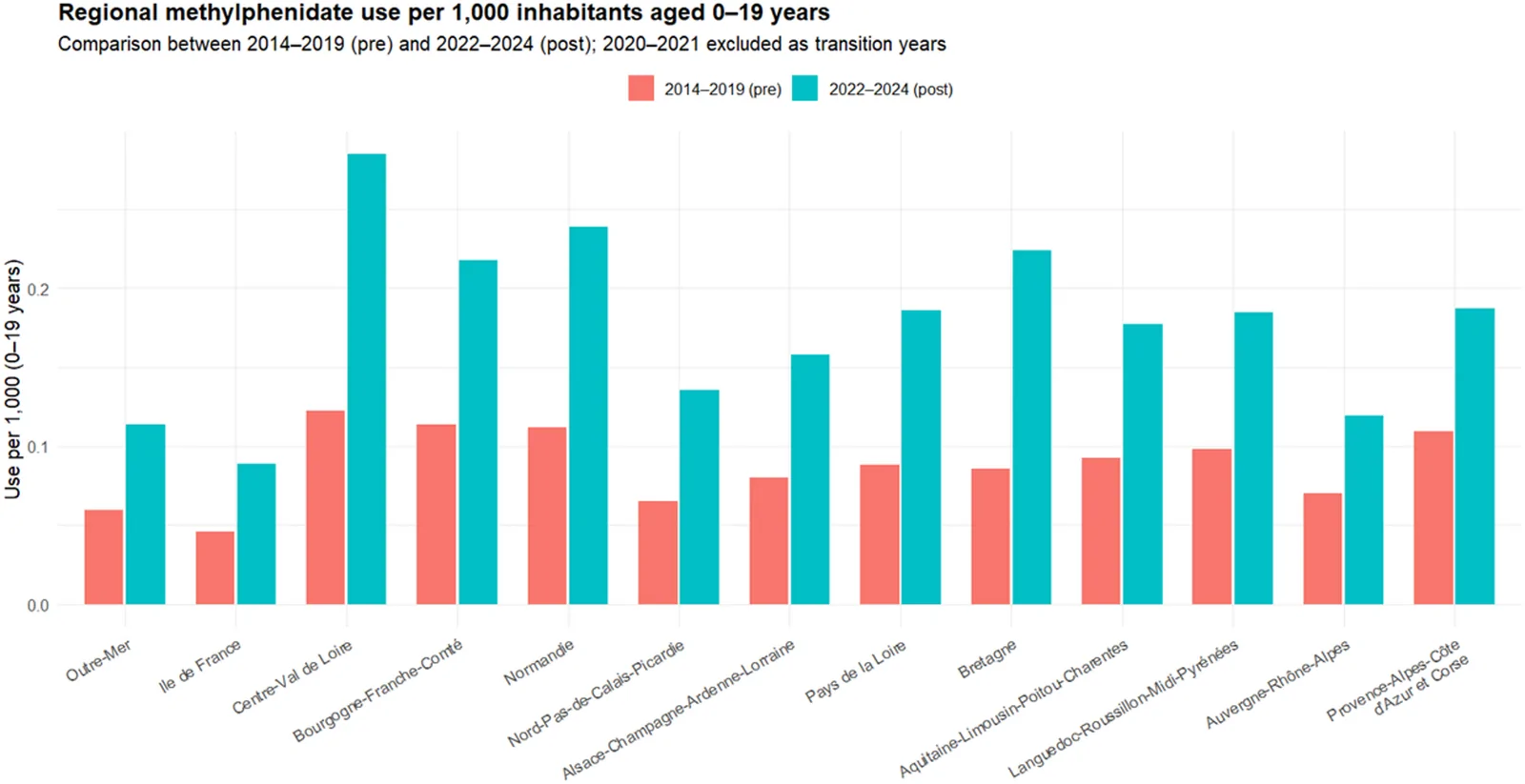
Regional methylphenidate dispensations per 1,000 inhabitants aged 0–19 years in France, 2014–2019 vs. 2022–2024 (2020–2021 excluded). Regional methylphenidate dispensations per 1,000 inhabitants aged 0-19 years were aggregated over the 2014-2019 period vs the 2022-2024 period. Transition years 2020-2021 are excluded

**Fig. 2:**
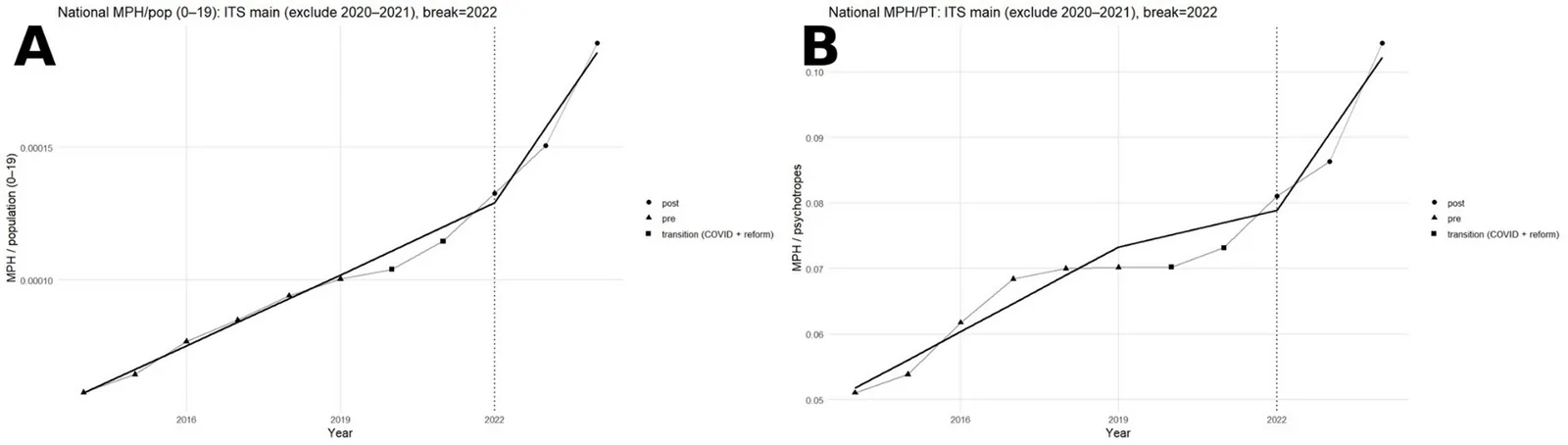
National interrupted time-series (ITS) models excluding 2020–2021 and fixing the breakpoint at 2022. **A **MPH dispensations divided by the population aged 0–19 years. **B ** MPH dispensations divided by all psychotropic dispensations. Points show observed annual national values; the solid line shows fitted values from the segmented regression for the main ITS model; the vertical dotted line marks the breakpoint year (2022). Observations from 2020-2021 are shown for reference but excluded from model estimation

Analyses were prespecified and organized as primary, secondary, and exploratory components. Given the ecological design, the annual aggregation of the data, and the co-occurrence of the 2021 reform with the COVID-19 period and other changes in ADHD care, all analyses were interpreted descriptively and were not intended to isolate the causal effect of the reform. In the main text, we report a parsimonious set of analyses addressing the study objectives: (i) regional descriptive pre/post comparisons (Fig. 1), (ii) national annual series with main ITS descriptions using a pre-specified 2022 breakpoint and exclusion of 2020–2021 (Fig. 2), and (iii) pre/post comparisons of MPH/PT and regional inequality metrics (Table 1).

Region-year fixed-effects ITS estimates (Table S1), pre-period window sensitivity analyses (Table S2), exploratory segmented breakpoint estimation (Figure S1), and exploratory structural correlations (Table S3) were considered as complementary analyses and are reported in the Supplementary Appendix.

### Primary analyses

#### Regional descriptive comparison

For each region, we summarized methylphenidate dispensations per 1,000 inhabitants aged 0–19 years in the pre-period (2014–2019) and post-period (2022–2024). Period-specific rates were computed by aggregating dispensations and population denominators across years within each period. Regional period values were displayed graphically (Fig. 1).

#### National annual series and national ITS description

National annual series were constructed by summing dispensations and population counts across the 13 regions for each year (2014–2024), then computing the national methylphenidate rate per 1,000 (0–19 years) and national MPH/PT. We then fit national interrupted time-series (ITS) models using annual observations from 2014 to 2019 and 2022–2024 only, with a pre-specified breakpoint at 2022 and excluding 2020 and 2021. The calendar year was centered at 2014 (t = year − 2014) to make intercepts interpretable as the expected outcome level in 2014. For each outcome, $\:{Y}_{t}$, we fit

$$\:{Y}_{t}={\beta\:}_{0}+{\beta\:}_{1}t+{\beta\:}_{2}Pos{t}_{t}+{\beta\:}_{3}{t}_{after,t}+{\epsilon\:}_{t},$$.

where $\:Pos{t}_{t}=1$ for years ≥ 2022 (0 otherwise) and $\:{t}_{after,t}=\mathrm{m}\mathrm{a}\mathrm{x}(0,year-2022)$. In this parameterization, $\:{\beta\:}_{1}$ represents the pre-period slope, $\:{\beta\:}_{2}$ the level difference at 2022, and $\:{\beta\:}_{3}$ the post-2022 change in slope (post-period slope = $\:{\beta\:}_{1}+{\beta\:}_{3}$). Because the national annual series includes few time points, and each observation aggregates dispensations and denominators for a large population, these national models were used to describe temporal patterns, and any inferences were interpreted cautiously (Fig. 2).

### Secondary analyses

#### Pre-post comparisons for MPH/PT

For national pre/post comparisons of MPH/PT (2014–2019 vs. 2022–2024), we used a two-sample test for equality of proportions based on aggregated counts. To examine whether changes differed across regions, we fitted binomial models using aggregated counts (MPH vs. other psychotropics), including region, period, and their interaction, and evaluated the region × period interaction using a likelihood-ratio test (Table 1).

#### Inequality indices

To quantify regional disparities in methylphenidate use relative to all psychotropics (MPH/PT), we adapted the WHO Health Equity Assessment Toolkit (HEAT) framework [53], and reported both absolute and relative inequality measures (Table 1). Absolute inequality was summarized using the Difference (highest minus lowest regional value) and the population-weighted Between-Group Variance (BGV), which captures the dispersion of regional values around the national mean while weighting each region by its population size. BGV is non-negative, with higher values indicating greater inequality and zero indicating perfect equality. As noted by Schlotheuber and Hosseinpoor [53], BGV is sensitive to outliers because regions that deviate strongly from the national average contribute disproportionately through squared deviations. We also reported the Between-Group Standard Deviation (BGSD), defined as the square root of BGV and expressed on the same scale as the outcome. Relative inequality was assessed using the Ratio (highest/lowest regional value) and the Gini coefficient derived from the Lorenz curve (0 indicates perfect equality and 1 indicates maximal inequality). Values below 0.1 are generally interpreted as indicating relatively low inequality in population health applications. To ensure comparability across periods, population weights from the 2014–2019 reference period were held constant when computing inequality metrics for both 2014–2019 and 2022–2024. 95% confidence intervals and between-period differences in inequality indices were estimated using non-parametric bootstrap resampling with 10,000 region-level resamples, with weights re-normalized within each resample (Table 1).

#### Panel region-year fixed-effects ITS

To leverage the region-year panel (13 regions observed over 2014–2019 and 2022–2024), we fit region fixed-effects models with the same ITS structure:

$$\:{Y}_{rt}={\beta\:}_{1}t+{\beta\:}_{2}Pos{t}_{t}+{\beta\:}_{3}{t}_{after,t}+{\alpha\:}_{r}+{\epsilon\:}_{rt},$$

where $\:{\alpha\:}_{r}$ denotes region fixed effects. We used cluster-robust standard errors by regions. Models were estimated without weights and with analytic weights equal to the regional population aged 0–19 years. Pre- and post-period slopes and 95% confidence intervals were derived from the model variance-covariance matrix using linear combinations of coefficients (delta method). To reduce reliance on breakpoint estimation with few annual observations, breakpoint estimation was not used in these panel models; instead, the breakpoint was fixed a priori at 2022. Estimates are reported in Table S1. Sensitivity to the choice of the pre-period window is reported in Table S2.

### Exploratory analyses

#### Exploratory segmented breakpoint estimation

As an exploratory analysis, we fit a segmented regression to the national annual methylphenidate rate per 1,000 over 2018–2024, allowing the breakpoint to be estimated from the data. This analysis included 2020–2021 and was treated as illustrative, given the limited number of annual observations and the potential influence of pandemic-related disruptions on the estimated breakpoint and its uncertainty (Figure S1). We additionally report the estimated breakpoint with its standard error and an approximate Wald-type 95% confidence interval to describe location uncertainty; these quantities are presented descriptively and were not used for confirmatory inference.

#### Exploratory structural correlations

Beyond national trends and aggregate inequality indices, we conducted an exploratory correlation analysis (Table S3) to examine potential structural correlates of regional differences using aggregated regional data (k = 12), excluding the overseas region (“Outre-mer”) because FDep15 was unavailable. Two indicators of methylphenidate use were analyzed: (a) methylphenidate dispensations per 1,000 inhabitants aged 0–19 years, and (b) the absolute number of methylphenidate dispensations. For each indicator, Pearson correlations were computed over the overall period (pre and post combined) and separately for the pre-period (2014–2019) and post-period (2022–2024), using two-tailed tests for the null hypothesis of zero correlation.

Contextual variables included psychiatrist density and child psychiatrist density (per 1,000 inhabitants at the regional level; source: France’s National Register of Health Professionals [CNOM], 2023) [54]. Socioeconomic conditions were captured using the French deprivation index (FDep15), a validated indicator of socioeconomic disadvantage (Santé Publique France and CepiDC-Inserm) [55]. FDep15 combines four weighted components: median tax income per consumption unit, the proportion of baccalaureate-level graduates among individuals aged ≥ 15 years who are not attending school, the proportion of labor workers in the working population aged 15–64 years, and the proportion unemployed in the working population aged 15–64 years. Higher values indicate greater deprivation. Regional FDep15 scores were derived from IRIS-level data (“Ilots Regroupés pour l’Information Statistique”, approximately 10,000 inhabitants each) and aggregated to the regional level using population weights. Economic context was characterized using regional gross domestic product (GDP) per capita in 2023 (INSEE) [56], defined as total regional production of goods and services divided by the number of inhabitants. Given the limited number of regions, these correlation analyses were interpreted descriptively (Table S3).

### Software

All analyses were conducted at the ecological level using reproducible R scripts (see Code availability). Data were managed in Microsoft Excel and analyzed in R (RStudio Version 2025.09.1 + 401).

## Results

### Regional patterns (2014–2019 vs. 2022–2024)

Across the 13 French regions, methylphenidate (MPH) dispensations per 1,000 inhabitants aged 0–19 years were higher in 2022–2024 than in 2014–2019 (Fig. 1). The range across regions increased from 0.046 to 0.122 per 1,000 in 2014–2019 to 0.089–0.285 per 1,000 in 2022–2024, with Île-de-France consistently among the lowest and Centre-Val de Loire among the highest (Fig. 1).

### National time series and main interrupted time-series (ITS)

Nationally, MPH dispensations per 1,000 inhabitants aged 0–19 increased from 0.058 in 2014 to 0.100 in 2019, and further to 0.133 in 2022 and 0.189 in 2024, corresponding to a 3.3-fold increase between 2014 and 2024 (Fig. 2A). In the main ITS models excluding 2020–2021 and fixing the breakpoint at 2022, the fitted trajectories were consistent with a steeper post-2022 slope than the pre-2020 slope, while the estimated immediate level change at 2022 was imprecise (Fig. 2).

### MPH as a share of all psychotropics and inequality indices

The national proportion of MPH among all psychotropic dispensations (MPH/PT) increased from 6.30% in 2014–2019 to 9.09% in 2022–2024 (Table 1). Increases were observed across all regions (Table 1), and a likelihood-ratio test of the region × period interaction did not support strong heterogeneity in changes across regions (χ² [12] = 13.69, *p* = 0.321). Regional inequality indices for MPH/PT showed limited change between periods (Table 1).

The absolute difference (highest minus lowest region) increased from 2.24% to 3.63%, and the highest-to-lowest ratio changed from 1.42 to 1.47. Population-weighted between-group variance (BGV) increased from 0.00003660 (95% CI: 0.00001022–0.00005908) to 0.00007407 (95% CI: 0.00001919–0.00012480), with a bootstrap difference of 0.00003747 (95% CI: -0.00000008-0.00007348). The Gini coefficient was similar across periods (0.059713 vs. 0.058784; Δ=-0.000929, 95% CI: -0.015703-0.013173).

**Table 1:** Methylphenidate dispensations as a proportion of all psychotropic dispensations (MPH/PT) and indicators of regional inequality in France, 2014–2019 vs. 2022–2024

	2014–2019	2022–2024	Comparisons
	Proportion (%) MPH/ all psychotropics	Proportion (%) MPH/all psychotropics	
All areas	6.30%	9.09%	χ²(1) = 561,60, *p* < 0.001
Subnational regions			
Outre-Mer	6,43%	8.84%	χ²(12) = 13.69, *p* = 0.321
Ile de France	5,38%	7.68%	
Centre-Val de Loire	7,62%	11.31%	
Bourgogne-Franche-Comté	6,96%	9.08%	
Normandie	6,23%	9.34%	
Nord-Pas-de-Calais-Picardie	5,80%	8.69%	
Alsace-Champagne-Ardenne-Lorraine	5,97%	8.35%	
Pays de la Loire	6,45%	9.42%	
Bretagne	5,95%	10.04%	
Aquitaine-Limousin-Poitou-Charentes	6,22%	8.96%	
Languedoc-Roussillon-Midi-Pyrénées	6,62%	9.78%	
Auvergne-Rhône-Alpes	6,25%	8.74%	
Provence-Alpes-Côte d’Azur et Corse	7,33%	9.74%	
Indicators of subnational regional inequality			
Difference	2.24%	3.63%	Δ = 1.39
Ratio	1.42	1.47	Δ = 0.05
Between-group variance (BGV)	0.00003660 (95% CI 0.00001022 ; 0.00005908)	0.00007407 (95% CI 0.00001919 ; 0.00012480)	Δ = 0.00003747 (95% CI − 0.00000008 ; 0.00007348)
Between-group standard deviation (BGSD)	0.00604961 (95% CI 0.00319637 ; 0.00768649)	0.00860627 (95% CI 0.00438107 ; 0.01117146)	Δ = 0.00255666 (95% CI − 0.00000977 ; 0.00426469)
Gini coefficient	0.059713 (95% CI 0.028172 ; 0.075707)	0.058784 (95% CI 0.027974 ; 0.075933)	Δ = −0.000929 (95% CI − 0.015703 ; 0.013173)

Values are percentages unless otherwise indicated. National comparisons were assessed using a two-sample test for equality of proportions. Regional heterogeneity in changes over time was evaluated using a likelihood-ratio test of the region x period interaction from binomial models. Indicators of subnational inequality include the absolute difference (highest minus lowest region), ratio (highest/lowest), population-weighted between-group variance (BGV), between-group standard deviation (BGSD), and Gini coefficient, with 95% confidence intervals estimated by bootstrap resampling of regions (10,000 iterations)

### Panel region-year fixed-effects ITS

In region-year panel models (117 region-years; 2014–2019 and 2022–2024 only) with region fixed effects and cluster-robust standard errors, MPH/population (0–19) expressed per 1,000 showed a higher post-2022 slope than pre-2020 (Table S1). In unweighted models, the estimated pre slope was 0.010 per 1,000/year (95% CI: 0.008–0.012) and the post slope 0.032 per 1,000/year (95% CI: 0.025–0.039), corresponding to a slope ratio of 3.21; the estimated level change at 2022 was 0.003 per 1,000 (95% CI: -0.008 to 0.014). Population-weighted models yielded similar estimates (pre: 0.009; post: 0.028 per 1,000/year).

For MPH/PT, slopes increased from 0.448% points/year pre-period (95% CI: 0.389–0.507) to 1.178% points/year post-period (95% CI: 1.043–1.313) in unweighted models, with similar results in population-weighted models (Table S1). The post-period level term was negative in these models (e.g., -0.798% points; 95% CI: -1.266 to -0.329), while post-period slopes were higher than pre-period slopes.

Sensitivity analyses varying the start of the pre-period (2014–2017) while keeping the post-period fixed (2022–2024) showed stable post slopes for MPH/population (0–19) per 1,000, with modest variation in the estimated pre slope and imprecise level-change estimates (Table S2).

### Exploratory breakpoint estimation and correlations

In the exploratory segmented model (2018–2024), the estimated breakpoint was 2021.56 (SE 0.372), corresponding to a Wald-type 95% confidence interval of 2020.83-2022.29 (Figure S1), indicating substantial uncertainty in the breakpoint location across the pandemic/reform transition period. Given the inclusion of the pandemic years and the small number of annual observations, this estimate is presented as an illustrative summary of the observed time-series shape rather than as confirmatory evidence regarding the policy reform.

Exploratory regional correlations (*n* = 12 regions; Outre-mer excluded because FDep15 was missing) indicated that MPH dispensations per 1,000 (0–19) was inversely correlated with psychiatrist density (overall *r* = -0.578, *p* = 0.0489) and GDP per capita (overall *r* = -0.637, *p* = 0.026), while correlations with child psychiatrist density were near zero and correlations with deprivation (FDep15) were positive but not statistically significant. In contrast, using the absolute number of MPH dispensations, correlations were positive with psychiatrist density (overall *r* = 0.626, *p* = 0.029) and child psychiatrist density (overall *r* = 0.609, *p* = 0.036), and negative with deprivation (overall *r* = -0.598, *p* = 0.040)(Table S3).

## Discussion

### Interpretation of the main findings

Regional disparities persisted alongside a marked national increase in methylphenidate dispensing over the study period. In our data, overall dispensing intensity increased over time, while regional differences remained observable, suggesting that rising national use did not translate into clear regional convergence. Similar configurations have been described internationally [19, 57, 58], with territorial variation in ADHD medication use persisting over long periods despite changes in overall levels of use.

Overall, dispensation volumes increased markedly between the pre- and post-periods, with national population-standardized methylphenidate dispensing more than tripling between 2014 and 2024. In the descriptive national time-series and panel models, post-2022 slopes were steeper than pre-period slopes, suggesting an acceleration of the pre-existing upward trend, with 2022 used as a descriptive marker of the post-transition period rather than as evidence of an isolated reform effect. This pattern is consistent with international observations showing that ADHD pharmacotherapy often increases over time, sometimes followed by stabilization or declines in specific settings [2, 3, 14, 17–19]. French SNDS evidence reporting increasing psychotropic dispensations in youth [37] also suggests that mental health treatment demand and/or dispensing activity had already been rising before the reform period. The exploratory segmented regression of the national annual series (2018–2024, including 2020–2021) similarly suggested an inflection around 2021.6, although this estimate should be interpreted cautiously because of the inclusion of pandemic years, the co-occurrence of the reform and pandemic period, and the small number of annual observations.

The COVID-19 pandemic is an important contextual factor for interpreting the temporal patterns observed in this study, rather than only a statistical nuisance. International evidence suggests that the pandemic affected healthcare utilization, diagnostic pathways, follow-up practices, and psychotropic medication dispensing in heterogeneous ways across countries and periods, including disruptions, rebounds, and shifts in care organization such as telemedicine use and altered access to specialist services [41–43]. In this context, the observed inflection in national methylphenidate dispensing should be interpreted as occurring across an overlapping pandemic/reform transition period, rather than as evidence of a specific reform effect. This is why 2020 and 2021 were treated as transition years in the main pre/post and fixed-breakpoint analyses.

The absence of clear convergence across regions in the inequality metrics indicates that regional differences persisted throughout the study periods. This apparent paradox reflects the distinction between parallel increases and convergence: all regions may increase over time, while relative or absolute gaps persist if higher-dispensing regions also continue to increase, or if increases in lower-dispensing regions are not large enough to close the gap. Territorial gradients in specialist availability, service organization, and care capacity are longstanding features of the French mental health system [14]. In this context, the increase in national dispensing coexisted with persistent regional variation. Exploratory correlation analyses, interpreted descriptively, also suggested that regional dispensing indicators were associated with medical and socioeconomic characteristics, consistent with previous work showing that regional disparities in ADHD diagnosis and care are shaped by healthcare resources and socioeconomic conditions [29–31, 56]. When expressed as absolute numbers, methylphenidate dispensations were higher in wealthier regions and in areas with greater psychiatrist density, consistent with larger population size and higher overall healthcare activity. When standardized to the regional population aged 0–19 years, however, correlations with psychiatrist density and GDP per capita were negative. This contrast likely reflects the fact that absolute counts and population-standardized rates capture different dimensions of regional variation: the former are more closely related to population volume and healthcare activity, whereas the latter describe dispensing intensity relative to the population denominator.

Sensitivity analyses varying the start of the pre-period in the panel fixed-effects models yielded similar post-period slopes for methylphenidate dispensations per 1,000 inhabitants aged 0–19 years, with only modest variation in pre-period slopes and imprecise level-change estimates. Overall, the descriptive pattern was stable across alternative pre-period definitions, consistently showing steeper post-period slopes without a clear immediate level shift.

Moreover, it is important to distinguish international prevalence estimates [1–9], which reflect the proportion of children and adolescents receiving ADHD prescriptions, from the OPENMEDIC indicators used in this study. OPENMEDIC captures reimbursed dispensations of methylphenidate boxes in community pharmacies rather than patient-level treatment rates or new treatment initiations. These data complement international prevalence estimates by documenting real-world medication delivery, although they do not directly indicate the proportion of treated individuals, treatment incidence, or the initiation processes targeted by the 2021 reform. Because MPH/PT uses all psychotropic dispensations as the denominator, and psychotropic medication use also increased among French youth over this period, this relative indicator may understate the magnitude of the increase in methylphenidate dispensing when considered in absolute or population-standardized terms.

Importantly, higher methylphenidate dispensing should not be interpreted as straightforward evidence of improved overall ADHD care. Pharmacological treatment is only one component of ADHD management, and comprehensive care also includes psychoeducation, behavioral approaches, school-based support, and psychotherapeutic or family-oriented interventions, particularly for younger children and depending on clinical severity and context [10, 11, 31]. In France, Ponnou and Thomé [14] reported that educational and psychotherapeutic follow-up among children and adolescents receiving methylphenidate decreased from 4.1% in 2010 to 0.8% in 2019, while methylphenidate use increased over the same period. This apparent divergence highlights the need to distinguish access to medication from access to comprehensive, multimodal, and clinically appropriate ADHD care.

Beyond healthcare supply and macro-socioeconomic indicators, other contextual factors may also contribute to regional variation in stimulant dispensing. Developmental and social environments-such as early parent-child interactions, family stress, and cumulative social adversity-have been associated with the expression, persistence, and recognition of ADHD symptoms over time [29, 30]. Longitudinal evidence also suggests that negative parental responses during frustration-inducing situations may be linked to attentional and behavioral dysregulation through mechanisms such as delay aversion and to developmental trajectories of ADHD-related behaviors [59, 60]. These processes may influence whether symptoms are clinically recognized and subsequently treated, and could therefore contribute to population-level dispensing patterns, although such mechanisms could not be directly tested in our aggregated dataset. Institutional contexts, including child protection and out-of-home care, have likewise been associated with distinct diagnostic and prescribing practices, often characterized by higher psychotropic exposure and different medication profiles compared with the general population [61, 62]. Although these family-level, cultural, and institutional dimensions could not be examined in the present dataset, they may help explain why territorial inequalities in ADHD treatment persist despite broader regulatory change. Future research using individual-level longitudinal data linking social context, care pathways, and prescribing practices would help clarify their contribution.

To interpret the findings of this study, it is also worth situating them within broader international frameworks. In the United Kingdom, methylphenidate is classified as a Schedule 2 controlled drug. According to NICE guidelines (NG87, 2018) [10], the treatment must be initiated by a clinician experienced in ADHD diagnosis and management (psychiatrist, pediatrician, or trained physician), after which it can be continued in primary care under a shared care arrangement. In Denmark, methylphenidate prescriptions are only permitted following specialized assessment and combined with psychoeducational interventions. National guidelines published in 2021 [23] specify that medication should be reserved for severe cases after non-pharmacological treatments have failed. In the United States, methylphenidate is classified as a Schedule II controlled substance under the Controlled Substances Act [24]. Prescriptions cannot be automatically renewed; however, physicians can issue sequential prescriptions that cover up to 90 days. Since the COVID-19 pandemic, federal authorities have extended allowances for prescribing controlled substances via telemedicine until the end of 2026. In mainland China, methylphenidate is regarded as a narcotic with strict national regulations controlling its prescription and distribution [25, 26]. These international comparisons illustrate that national regulatory frameworks, service organization, and prescribing rules are likely to be associated with both access to ADHD treatment and territorial variation in its use. In France, this issue is particularly salient, as mental health was designated a “*Grande Cause Nationale”* in 2025 and 2026 [63, 64], reflecting a strong political commitment to reducing inequalities in psychiatric care, including in child and adolescent psychiatry. Against this policy backdrop, our findings describe an increase in overall methylphenidate dispensing over the study period, alongside persistent regional disparities. They therefore provide a descriptive account of trends occurring in the context of regulatory change, pandemic-related disruptions, and broader changes in ADHD care, rather than evidence that the 2021 reform itself produced measurable convergence in regional inequality metrics.

### Strengths and limitations

This study draws strength from the use of a large, nationwide database providing exhaustive coverage of reimbursed outpatient dispensations in France. The OPENMEDIC dataset includes annual data on methylphenidate and psychotropic dispensations for individuals aged 0–19 years, and these data align directly with INSEE population estimates using the same age range and regional structure. Another strength is that the main descriptive pattern - namely steeper post-period slopes in the fixed-breakpoint models - was consistent across weighted and unweighted panel fixed-effects specifications and across alternative pre-period definitions, supporting the stability of the observed pattern within the constraints of annually aggregated ecological data.

This study has several limitations. First, its ecological design and reliance on annually aggregated data precluded assessment of individual-level variability, clinical trajectories, seasonality, and short-term changes in prescribing. The years 2020 and 2021 were treated as transition years in the main analyses because they captured overlapping pandemic-related disruptions and implementation of the 2021 reform. However, the reform period also overlapped with broader changes in ADHD care, making it difficult to isolate the contribution of any single measure. The steady increase in methylphenidate dispensing observed before 2021 also suggests that part of the post-2022 acceleration may reflect continuation of pre-existing trends rather than a wholly new change associated with the reform period.

Second, OPENMEDIC records dispensed boxes rather than treated individuals. Accordingly, our indicators reflect dispensing intensity rather than patient-level treatment, and the database does not allow distinction between new treatment starts, continuation or renewal, despite the fact that the 2021 reform specifically concerned initiation. Therefore, this study cannot directly evaluate whether the reform changed treatment initiation practices. In addition, the dataset does not provide information on dosage, formulation, pack size, or days supplied, and box counts may therefore be influenced by changes in dispensing practices or supply constraints over time. It also does not provide information on clinical pathways, shared-care arrangements, continuity of follow-up, access to non-pharmacological interventions, treatment appropriateness, or clinical outcomes after initiation. Finally, because publicly available data were aggregated for the 0-19-year age group, the denominator included children younger than 6 years, for whom methylphenidate is not licensed in Europe [12]. This likely diluted absolute dispensing rates and prevents age-specific interpretation of treatment intensity among clinically eligible age groups.

Third, our analyses describe methylphenidate dispensations specifically and do not capture possible substitutions with other ADHD medications, including stimulants and non-stimulants, which may vary across settings and over time. Finally, the small number of regional units (*k = 13*) limited statistical power, particularly for detecting modest between-period changes in regional inequality metrics, and therefore required a cautious interpretation of exploratory structural correlations.

### Implications

Our findings describe an increase in overall methylphenidate dispensing in France during the study period, while regional disparities persisted. This pattern suggests that changes in prescribing rules, when occurring within broader pandemic-related and organizational changes, may coexist with longstanding structural imbalances rather than necessarily producing regional convergence. The magnitude and pace of this increase also raise important questions about treatment appropriateness, diagnostic assessment, monitoring of potential adverse effects, and the capacity of services to provide longitudinal follow-up and multimodal care alongside medication. Although methylphenidate dispensing levels in France remain lower than those reported in the United States and other high-prescribing countries, the rapid increase observed over a short period warrants careful public health attention. In this context, reducing territorial disparities in ADHD care may require broader and more coordinated public health actions beyond prescribing rules, including targeted investments in psychiatric workforce capacity and service availability, as well as professional training in up-to-date evidence-based ADHD assessment and management and, where relevant, culturally informed or trauma-informed approaches [34].

From a research perspective, access to more granular data sources would enable a finer assessment of individual-level prescribing patterns, interregional disparities, and temporal associations with policy changes. Future studies using monthly or patient-level data could better characterize the timing of observed changes, distinguish treatment initiation from continuation or renewal, and help assess the relative contribution of the 2021 reform, pandemic-related disruptions, and broader changes in mental health care utilization. At an international level, these findings illustrate how national prescribing regulations and service organization may be associated with access to pharmacological ADHD treatment, while equity in comprehensive ADHD care also depends on access to diagnostic assessment, psychosocial interventions, follow-up, and broader service capacity. Our findings also complement recent evidence [65] showing that key sociodemographic factors remain poorly reported in ADHD medication trials, suggesting that the regional disparities observed here may reflect both differences in access to care and the underrepresentation of vulnerable populations in the literature. Comparative studies across countries [31] could help inform policies aimed at promoting equitable access to stimulant medications within broader child and adolescent mental health systems. Overall, these results support the idea that changes in prescribing regulation may coexist with persistent structural inequities in access to care, a challenge shared across health systems.

## Conclusion

This nationwide ecological analysis based on annually aggregated OPENMEDIC and INSEE data found that methylphenidate dispensing in France more than tripled between 2014 and 2024, with steeper post-2022 trends, whereas regional disparities persisted and inequality metrics showed no clear convergence. Because the 2021 prescription reform coincided with the COVID-19 period and broader changes in ADHD care, these findings should be interpreted as descriptive population-level trends rather than evidence of a specific reform effect. Reducing territorial inequalities in ADHD care will likely require broader structural actions beyond changes in prescribing rules alone.

## Supplementary Information

Supplementary Material 1.

Supplementary Material 2.

Supplementary Material 3.

Supplementary Material 4.

## Data Availability

All data used in this study are publicly available from the French National Health Insurance (OPENMEDIC: [https://assurance-maladie.ameli.fr/etudes-et-donnees/open-medic-base-complete-depenses-medicaments](https:/assurance-maladie.ameli.fr/etudes-et-donnees/open-medic-base-complete-depenses-medicaments) ) and the French National Institute of Statistics and Economic Studies (INSEE: [https://www.insee.fr/fr/statistiques/8331297](https:/www.insee.fr/fr/statistiques/8331297) ). The datasets analyzed during the current study are fully anonymized and aggregated at the regional level.

## Code availability

All custom R scripts used for data preprocessing, descriptive analyses, interrupted time-series analyses, inequality metrics, bootstrapped confidence intervals, chi-square analyses, and the generation of all tables and figures reported in this manuscript have been deposited on Zenodo and are openly accessible at: https://doi.org/10.5281/zenodo.19019051.

Readers should refer to version 2 of the Zenodo record. The archive contains fully annotated code and all functions necessary to reproduce the analyses, tables, and figures reported in this manuscript

## Abbreviations

ADHD: Attention-deficit/hyperactivity disorder
ANSM: Agence Nationale de Sécurité du Médicament et des produits de santé / French National Agency for the Safety of Medicines and Health Products
ATC: Anatomical Therapeutic Chemical classification
BGV: Between-group variance
BGSD: Between-group standard deviation
CNOM: Conseil National de l’Ordre des Médecins / National Register of Health Professionals
FDep15: French deprivation index
GDP: Gross domestic product
HEAT: Health Equity Assessment Toolkit
INSEE: Institut National de la Statistique et des Etudes Economiques / National Institute of Statistics and Economic Studies
IRIS: Ilots Regroupés pour l'Information Statistique / French submunicipal statistical units
ITS: Interrupted time-series
MPH: Methylphenidate
PT: Psychotropic dispensations
SNDS: Système National des Données de Santé / French National Health Data System
