# Supplementary material for: Trends in methylphenidate dispensing and regional inequalities among children and adolescents in France around the 2021 prescription reform: a repeated cross-sectional ecological analysis of national data (2014–2024)
Source: BMC Public Health. 2026 Jun 26;26:2583. doi: 10.1186/s12889-026-28213-3 (PMC13563797; doi:10.1186/s12889-026-28213-3)
Supplement: Supplementary file 1 [file 12889_2026_28213_MOESM1_ESM.docx]

**Figure S1.** Segmented regression of national MPH dispensations divided by the population aged 0-19 years over 2018-2024.

***Note.*** *The dotted vertical line marks the estimated breakpoint (approximately 2021.6). The shaded band represents the 95% confidence interval of the fitted segmented trend.*
