# Supplementary material for: Trends in methylphenidate dispensing and regional inequalities among children and adolescents in France around the 2021 prescription reform: a repeated cross-sectional ecological analysis of national data (2014–2024)
Source: BMC Public Health. 2026 Jun 26;26:2583. doi: 10.1186/s12889-026-28213-3 (PMC13563797; doi:10.1186/s12889-026-28213-3)
Supplement: Supplementary file 2 [file 12889_2026_28213_MOESM2_ESM.docx]

**Table S1.** Panel region-year fixed-effects ITS estimates (2014-2019 and 2022-2024; excluding 2020-2021; breakpoint fixed at 2022).

| **Outcome** | Specification | Pre slope (95% CI) | Post slope (95% CI) | Slope ratio | Level change (95% CI) | Notes |
| --- | --- | --- | --- | --- | --- | --- |
| MPH/pop (0-19) per 1,000 | Unweighted | 0.010 (0.008 to 0.012) | 0.032 (0.025 to 0.039) | 3.21 | 0.003 (-0.008 to 0.014) | Rates expressed per 1,000 (0-19) |
| MPH/pop (0-19) per 1,000 | Population-weighted | 0.009 (0.007 to 0.011) | 0.028 (0.022 to 0.035) | 3.16 | 0.001 (-0.007 to 0.008) | Rates expressed per 1,000 (0-19) |
| MPH/PT (proportion) | Unweighted | 0.448 (0.389 to 0.507) | 1.178 (1.043 to 1.313) | 2.63 | -0.798 (-1.266 to -0.329) | Slopes in percentage points/year |
| MPH/PT (proportion) | Population-weighted | 0.423 (0.375 to 0.472) | 1.147 (1.042 to 1.251) | 2.71 | -0.744 (-1.041 to -0.448) | Slopes in percentage points/year |

***Note.*** *Models include region fixed effects and cluster-robust standard errors by region. Population-weighted models use the regional population aged 0-19 years as analytical weights*
