# Supplementary material for: Trends in methylphenidate dispensing and regional inequalities among children and adolescents in France around the 2021 prescription reform: a repeated cross-sectional ecological analysis of national data (2014–2024)
Source: BMC Public Health. 2026 Jun 26;26:2583. doi: 10.1186/s12889-026-28213-3 (PMC13563797; doi:10.1186/s12889-026-28213-3)
Supplement: Supplementary file 3 [file 12889_2026_28213_MOESM3_ESM.docx]

## **Table S2.** Sensitivity of the panel fixed-effects estimates for MPH/population (0-19) to the choice of the pre-period window start year (post period fixed at 2022-2024; excluding 2020-2021).

| **Pre-period** | Post-period | Pre slope (95% CI) | Post slope (95% CI) | Level change (95% CI) |
| --- | --- | --- | --- | --- |
| 2014-2019 | 2022-2024 | 0.010 (0.008 to 0.012) | 0.032 (0.025 to 0.039) | 0.003 (-0.008 to 0.014) |
| 2015-2019 |  | 0.010 (0.008 to 0.012) | 0.032 (0.025 to 0.039) | 0.003 (-0.008 to 0.014) |
| 2016-2019 |  | 0.009 (0.007 to 0.011) | 0.032 (0.025 to 0.039) | 0.008 (-0.004 to 0.019) |
| 2017-2019 |  | 0.008 (0.006 to 0.010) | 0.032 (0.025 to 0.039) | 0.010 (-0.006 to 0.027) |

***Note.*** *Slopes and level changes are expressed per 1,000 inhabitants aged 0-19.*
