# Supplementary material for: Trends in methylphenidate dispensing and regional inequalities among children and adolescents in France around the 2021 prescription reform: a repeated cross-sectional ecological analysis of national data (2014–2024)
Source: BMC Public Health. 2026 Jun 26;26:2583. doi: 10.1186/s12889-026-28213-3 (PMC13563797; doi:10.1186/s12889-026-28213-3)
Supplement: Supplementary file 4 [file 12889_2026_28213_MOESM4_ESM.docx]

**Table S3.** Correlations between methylphenidate indicators and regional structural factors (n = 12 regions).

| **Variable** | **r for overall period (2014-2024)** | ***p*-value** | **r for the 2014-2019 period** | ***p*-value** | **r for the 2022-2024 period** | ***p*-value** |
| --- | --- | --- | --- | --- | --- | --- |
| **MPH dispensations per 1,000 inhabitants aged 0-19 years** | | | | | | |
| **FDep15 (deprivation index)** | 0.483 | 0.112 | 0.478 | 0.116 | 0.464 | 0.128 |
| **Psychiatrist density** | -0.578 | 0.0489 | -0.537 | 0.0721 | -0.586 | 0.045 |
| **Child psychiatrist density** | -0.100 | 0.7570 | 0.004 | 0.991 | -0.183 | 0.570 |
| **GDP per capita (2023)** | -0.637 | 0.026 | -0.657 | 0.020 | -0.589 | 0.044 |
| **Absolute number of MPH dispensations** | | | | | | |
| **FDep15 (deprivation index)** | -0.598 | 0.040 | -0.550 | 0.064 | -0.641 | 0.025 |
| **Psychiatrist density** | 0.626 | 0.029 | 0.599 | 0.040 | 0.642 | 0.025 |
| **Child psychiatrist density** | 0.609 | 0.036 | 0.606 | 0.037 | 0.590 | 0.043 |
| **GDP per capita (2023)** | 0.562 | 0.057 | 0.505 | 0.094 | 0.620 | 0.032 |

## *Note. Pearson correlation coefficients (r) were computed between regional methylphenidate indicators and structural contextual factors across 12 French regions ("Outre-mer" was excluded because of missing FDep15 values). Correlations were calculated for 2014-2019 period and 2022-2024 period, excluding 2020-2021. For each structural variable (psychiatrist density, child psychiatrist density, FDep15 deprivation index, and regional GDP per capita), r and its associated p-value were estimated using two-tailed Pearson tests.*
